# Hepatitis B vaccination coverage and serological status among health care workers exposed to occupational biological hazards

**DOI:** 10.47626/1679-4435-2022-963

**Published:** 2023-11-24

**Authors:** Fernanda Sucasas Frison, Herling Gregorio Aguilar Alonzo, Inajara de Cassia Guerreiro, Elaine Cristina Paixão de Oliveira

**Affiliations:** 1 Departamento de Saúde Coletiva, Universidade Estadual de Campinas (Unicamp), Campinas, SP, Brazil; 2 Centro de Saúde da Comunidade, Unicamp, Campinas, SP, Brazil

**Keywords:** occupational exposure, hepatitis B vaccines, hepatitis B antibodies, health personnel, acidentes de trabalho, vacinas contra hepatite B, anticorpos anti-hepatite B, trabalhadores da saúde

## Abstract

**Introduction:**

Health care workers are often exposed to hepatitis B infection during the course of their professional roles.

**Objectives:**

To analyze the hepatitis B vaccination coverage and the presence of antibodies against hepatitis B among health care professionals who were exposed to contaminated biological material at a hospital complex.

**Methods:**

This descriptive, retrospective, and quantitative study is based on the analysis of accident notification form data (n = 2,466) from a hospital complex covering the period between 2011 and 2020.

**Results:**

Among the affected individuals, women (69.5%), medical residents (35.7%), and nursing staff (25.5%) accounted for the highest proportion of hazards. Regarding vaccination status, 98% of the health care professionals reported being fully immunized, and antibodies were detected in 90.9% of the participants. Percutaneous exposure (76.4%) was the most prevalent type of hazard, with blood being the most commonly involved material (79.4%).

**Conclusions:**

The findings show that despite the risks of Hepatitis B contamination associated with the incidents, the professionals were protected due to the high vaccination coverage and evidence of immunity.

## Abstract (pt)

**Introdução:**

Os trabalhadores da saúde estão constantemente expostos ao vírus da hepatite B durante a atividade laboral.

**Objetivos:**

Analisar a cobertura vacinal contra a hepatite B e a presença do anticorpo contra o antígeno de superfície da hepatite B (anti-HBs) entre profissionais e estudantes da área da saúde que sofreram acidente com material biológico em um complexo hospitalar universitário.

**Métodos:**

Tratou-se de um estudo descritivo, retrospectivo e quantitativo, baseado na análise dos dados das fichas de notificação (n = 2.466) dos acidentes ocorridos no período de 2011 a 2020.

**Resultados:**

As mulheres (69,5%), os residentes de medicina (35,7%) e os técnicos e auxiliares de enfermagem (25,5%) foram os que mais se acidentaram. Quanto ao estado vacinal dos trabalhadores de saúde para hepatite B, 98% declararam ter o esquema vacinal completo, e a presença de anti-HBs reagente foi detectada em 90,9%. Quanto às características dos acidentes, houve prevalência de exposição percutânea (76,4%), e sangue foi o material orgânico mais comumente envolvido (79,4%).

**Conclusões:**

Os achados demonstram que, apesar do risco de contaminação para o vírus da hepatite B associado a acidentes no ambiente de trabalho, os profissionais estavam protegidos devido à elevada cobertura vacinal e à imunidade comprovada.

## INTRODUCTION

The Hepatitis B virus (HBV) represents a significant public health problem worldwide, given the substantial population of carriers of the etiologic agent, estimated at approximately 257 million chronically infected individuals. This condition increases the risk of liver-disease mortality and impairs the quality of life of those affected, while also imposing a considerable financial burden on the public healthcare system.^1,2^

In Brazil, between 1999 and 2020, a total of 254,389 confirmed cases of HBV infection were reported, with a higher prevalence observed in the Southeast and South regions.^3^ The country’s detection rate in 2020 was 2.9 cases per 100,000 inhabitants, indicating a declining trend since 2014.^3^ Over the period from 2000 and 2019, the Mortality Information System recorded a total of 78,642 deaths associated with viral hepatitis, 21.3% of which were related to HBV.^3^

Hepatitis B is distinguished from other types of viral hepatitis by its high transmissibility and the different routes of infection, which include vertical transmission, sexual intercourse, sharing needles or syringes, and exposure to contaminated needles or other sharp instruments that breach the skin barrier.^4^

Any individual can be exposed to HBV; however, there are groups at higher risk. These high-risk populations include hemodialysis patients, newborns born to infected mothers, men who have sex with men, sex workers, people who inject drugs, as well as health and public safety workers.^4^

The prevalence of HBV infection among health care workers (HCWs) has been reported to be up to four times higher than that in the general population.^5^ Accidents occurring during work are considered occupational hazards and are linked to the work environment, conditions, and organization. In occupational incidents where workers are exposed to biological material - occupational biological hazards (OBH), there is a risk of contamination during patient care, with potential exposure through needle sticks or other injuries contaminated with blood or body fluids on mucous membranes and non-intact skin.^5^

It is important to highlight that accidental transmission of human immunodeficiency virus (HIV), HBV, and hepatitis C virus (HCV) can occur as a result of OBHs. Consequently, a thorough assessment is necessary, considering factors such as the type of exposure, the extent and severity of the injury, the causative agent, and the serologic status of the source case.^6^

The risk of hepatitis B infection following percutaneous exposure to contaminated sharps ranges from 6 to 31%.^6^ In the event of an OBH, the incident must be reported to Social Security and a prompt notification to the Notifiable Diseases Information System (Sinan) must be submitted.^7^

Vaccination against HBV is the main measure of individual and collective protection, providing an effective, safe, and cost-free preventive approach. It has been included in the childhood vaccination schedule since 1998 and has been recommended for the entire population since 2016.^8^ Immunization consists of administering three intramuscular doses of the vaccine, following a schedule of 0, 1, and 6 months. In healthy individuals, the vaccine demonstrates an efficacy rate ranging from 90 to 95%.^8^

HCWs who have completed the HBV vaccination schedule and obtained a reactive hepatitis B surface antigen (HBs) antibody test result of 10 mIU/mL or higher are considered to be protected against infection.^1,4,5^ The verification of anti-HBs to demonstrate vaccine protection among HCWs is a measure recommended by the Brazilian Ministry of Health,^9^ and proof of vaccination is required during the hiring process in both public and private institutions. In cases where HCWs undergo OBHs with a positive source case for HBV but without reactive anti-HBs values, prophylaxis with hyperimmune immunoglobulin against hepatitis B is indicated.^10^

In Brazil, anti-HB testing after vaccination is not a routine practice in the public health system due to the high efficacy of the vaccine. Nevertheless, in specific situations, such as management after an OBH, this information becomes necessary.^10,11^

Regarding the prevention of infections from OBHs among HCWs, it is essential to adopt standard precautionary measures in addition to HBV vaccination. These measures include hand washing; proper disposal of instruments, chemical, and toxic waste; use of devices with retractable needles and needle protection systems; and the use of personal protective equipment (PPE) - gloves, isolation clothing, and face protection.^12^

Considering that vaccination is the primary measure for preventing occupational HBV infection,^1,5,8,12^ the present study aimed to analyze the vaccination coverage against hepatitis B and the presence of anti-HBs antibodies among health care professionals and students who underwent OBHs within a university hospital complex.

## METHODS

This was a descriptive and retrospective study of a case series, based on the notification forms of the OBHs that occurred between January 2011 and December 2020 in the hospital complex of Universidade Estadual de Campinas (Unicamp).

Unicamp is located in the countryside of the state of São Paulo, in the municipality of Campinas. The health area of the university comprises two hospitals, four specialized centers, and several outpatient clinics, where a wide range of highly complex procedures and activities related to teaching, research, and extension take place. Since 2011, HCWs exposed to OBHs within the institution have been treated and notified by the Biological Risk Program of the Community Health Center (CECOM), adhering to the protocols established by the Ministry of Health.^10^

The different professional categories and occupations were categorized as follows: physicians, medical residents, nurses, other higher education professionals (biomedical, biologists, dentists, physiotherapists, pharmacists, speech therapists, and psychologists), nursing technicians and assistants, health technicians (including necropsy, radiology, laboratory technicians, dental assistants, and laboratory assistants), undergraduate health students (medical, nursing, speech therapy, biology, biomedicine, and physiotherapy students), interns, and housekeeping personnel. We decided to separate the assistant physicians from the resident physicians because they have different links in the institution and in the practice of the profession.

To analyze vaccination status in relation to hepatitis B and serological status, the following variables were used: complete vaccination schedule (three doses or more); incomplete (less than three doses); unvaccinated; anti-HBs antibody titration (<10 mIU/mL for reactants and >10 mIU/mL for reactants); and HBV surface antigen (HBsAg) positive or negative in known source cases, i.e., patients who were involved in the accident, and in whom it was possible to collect the test.

In cases where the HCWs suffered more than one accident in the same year or over the study period, the first notification of each person was considered to assess vaccination status, selecting those who had non-reactive results for anti-HBs, and the need for HBV vaccine indication.

Regarding the characteristics of the OBH and the relationship with the vaccination status (complete or incomplete scheme), the following variables were evaluated: type of exposure, organic material involved; and use of PPE.

For the analyses, the selected data were included in a Microsoft Excel 2016 spreadsheet, and the following pieces of software were used: Statistical Packages for the Social Sciences version 20 and Minitab 16. The assessment of association was made using the chi-square or Fisher’s exact tests, and the test of equality of two proportions. In variables with three or more responses, the comparison was made with the most prevalent, which is marked in the tables as a reference (Ref). P-values less than 5% were considered statistically significant.

The ethical aspects of the research were respected, according to the guidelines of Resolution No. 466/2012 of the Brazilian National Health Council, and the Research Ethics Committee of the University approved the study under No. 3.510.458/2019.

## RESULTS

The total number of notification forms analyzed was 2,466; however, there was loss of data due to incomplete forms. As shown in Table 1, women were more prevalent, comprising 1,715 HCWs (69.5%), and among this group, 26 (1.5%) reported being pregnant at the time of the OBH.

**Table 1:** Distribution of demographic factors among health care professionals exposed to occupational biological hazards, 2011-2020, Hospital Area of Universidade Estadual de Campinas (n = 2,466)

Demographic factors	n	%	p-value
Age range (years)			
16 to 19	16	0.6	<0.001
20 to 29	1.262	51.2	Ref.
30 to 39	668	27.1	<0.001
40 to 49	338	13.7	<0.001
≥ 50	182	7.4	<0.001
Sex			
Women	1.715	69.5	<0.001
Men	751	30.5	
Color			
White	2.186	88.6	Ref.
Brown	145	5.9	<0.001
Black	74	3.0	<0.001
Yellow	53	2.1	<0.001
Not informed	8	0.3	<0.001
Pregnant			
No	1.683	98.5	<0.001
Yes	26	1.5	
Schooling			
Unknown	8	0.3	<0.001
Incomplete primary education	32	1.3	<0.001
Complete Elementary school	19	0.8	<0.001
Incomplete High school	29	1.2	<0.001
Complete High school	499	20.2	<0.001
Incomplete Higher education	364	14.8	<0.001
Complete Higher education	1.515	61.4	Ref.
Occupational activity			
Nurse	188	7.6	<0.001
Nursing technician/assistant	653	26.5	<0.001
Physician	162	6.6	<0.001
Medical resident	880	35.7	Ref.
Housekeeping personnel	93	3.7	<0.001
Health technician	67	2.7	<0.001
Graduate in Health	268	10.8	<0.001
Postgraduate in health	32	1.3	<0.001
Other professionals with higher education	91	3.7	<0.001
Internship	14	0.5	<0.001
Other	18	0.8	

Test of equality of two proportions.

Ref. = most prevalent variable.

The age of HCWs ranged from 16 to 70 years, with a mean of 32.6 years. Regarding self-reported skin color, a higher prevalence of white individuals was observed (2.186; 88.6%). Complete higher education was the predominant educational level (1.515; 61.4%), and medical residents (35.7%) reported the highest number of accidents among HCWs. Therefore, based on the data presented in Table 1, there is statistical significance in the analyzed demographic factors (p < 0.05).

Regarding the number of doses of hepatitis B vaccine at the time of accident notification, most (2,417; 98%) participants reported having three doses or more of the vaccine, 28 (1.2%) did not complete the vaccination schedule (less than three doses), 9 (0.3%) were not vaccinated, and 12 (0.5%) did not have this information.

Table 2 shows the analyses of the associations between hepatitis B vaccination (complete and incomplete schedule) and the profile of the OBH (type of exposure and organic material involved), the anti-HBs result (reactive or non-reactive), the use of PPE, and the professional category. A statistical relationship was observed between the hepatitis B vaccination schedule and the anti-HBs result, the organic material involved, and occupational activity. Regarding the relationship with the anti-HBs test, the non-reactive rate was 6% among the complete ones and 100% among those with incomplete schedules. On the other hand, the reactive rate was 94% among those with complete vaccination schedules and 0% among those with incomplete vaccination (p < 0.001).

**Table 2:** Distribution of health professionals exposed to biological material according to hepatitis B vaccination schedule by anti-HBs result, use of PPE, profession, and characteristics of the biological material, Hospital Area of Universidade Estadual de Campinas, 2011-2020

	Vaccination schedule						p-value
	Complete		Incomplete		Total		
	n	%	n	%	n	%	
Anti-HBs result							
Non-reactive	144	6.0	27	100.0	171	7.1	<0.001
Reactive	2,242	94.0	0	0.0	2,242	92.9	
Use of PPE							
No	258	11.1	1	3.7	259	11.0	0.223
Yes	2,071	88.9	26	96.3	2,097	89.0	
Organic material involved							
Formalin-fixed biopsy	10	0.4	1	3.6	11	0.4	<0.001
Ignored	99	4.1	8	28.6	107	4.4	
Clean or washed material	61	2.5	0	0.0	61	2.5	
Blood or bloodborne products	1,927	79.7	15	53.6	1,942	79.4	
High-risk secretion	54	2.2	0	0.0	54	2.2	
Low-risk secretion	156	6.5	3	10.7	159	6.5	
Sterile material	110	4.6	1	3.6	111	4.5	
Occupational activity							
Undergraduate student in the Health area	265	11.0	2	7.1	267	10.9	<0.001
Graduate student in the Health area	32	1.3	0	0.0	32	1.3	
Nurse	188	7.8	0	0.0	188	7.7	
Intern	14	0.6	0	0.0	14	0.6	
Physician	159	6.6	1	3.6	160	6.5	
Other higher education professionals	86	3.6	3	10.7	89	3.6	
Others	17	0.7	1	3.6	18	0.7	
Medical residents	873	36.1	5	17.9	878	35.9	
Housekeeping personnel	75	3.1	12	42.9	87	3.6	
Health technicians	60	2.5	3	10.7	63	2.6	
Nursing technicians/assistants	648	26.8	1	3.6	649	26.5	
Type of exposure							
Bite or scratch	12	0.5	0	0.0	1	0.5	0.953
Ocular mucosa	360	14.9	3	10.7	363	14.8	
Oral mucosa	36	1.5	0	0.0	36	1.5	
Oral and ocular mucosa	35	1.4	0	0.0	35	1.4	
Other	3	0.1	0	0.0	3	0.1	
Intact skin	66	2.7	1	3.6	67	2.7	
Non-intact skin	59	2.4	2	7.1	61	2.5	
Percutaneous	1,846	76.3	22	78.6	1,867	76.4	

Chi-square or Fisher’s exact test. Loss of data in all variables.

Anti-HBs = antibody against hepatitis B surface antigen; PPE = personal protective equipment.

Regarding the relationship between vaccination and occupational activity, all graduate students, nurses, and interns had a complete vaccination schedule. Among physicians, the rate was 6.6% among those with complete and 3.6% among those with incomplete schedules. Notably, although housekeeping professionals are not among the most frequent professions, they stand out with the most significant disparity, with a rate of 3.1% among those with complete vaccination and 42.9% among those with incomplete schedules.

Regarding the three characteristics of OBHs analyzed, among HCWs with complete and incomplete vaccination schedules, respectively, blood was the most frequently involved organic material in 79.7 and 53.6%, percutaneous exposure in 76.3 and 78.6%, and use of PPE, in 88.9 and 96.3%.

Anti-HBs serologic test values to prove immunity showed reactive results (>10 mIU/mL) in 2442 (90.9%) of the HCWS and non-reactive results (<10 mIU/mL) in 184 (7.47%) of the HCWS; this data was not available in 40 (1.63%) of the cases.

Considering only the first accident notification of each HCW, without recurrences, the total number of cases drops to 1,908. Among these, 152 had non-reactive results for anti-HBs (Table 3). Of these, 86 had a record of referral for vaccination, which was indicated by the professional responsible for the Biological Risk Program.

**Table 3:** Distribution of health care professionals exposed to biological material according to the indicated vaccines and vaccination schedule at the time of the accident, Hospital Area of Universidade Estadual de Campinas, 2011-2020 (n = 86)

	n	%	p-value
Indication			
With indication	86	56.6	0.022
No information on indication	66	43.4	
Indicated vaccines (hepatitis B)			
Fourth dose	65	75.6	Ref.
Intradermal scheme	8	9.3	<0.001
Third dose	7	8.1	<0.001
Second scheme	3	3.5	<0.001
Second dose	2	2.3	<0.001
Fifth dose	1	1.2	<0.001

Test of equality of two proportions.

Ref. = most prevalent variable.

According to Table 3, among the 86 cases with indication for vaccine, 75.6% had indication for the fourth dose, which represents a statistically significant difference compared to the other cases.

Regarding the source cases of OBHs, i.e. the patients involved in accidents where serological data were collected, 2.203 (89.4%) were known; the serological marker (HBsAg) tested positive in 15 cases (0.61%); and the presence of hepatitis B core antibody (anti-HBc), indicating current or past infection, was identified in 188 (7.6%) of the patients.

Three HCWs, one medical resident (with a complete vaccination schedule), one housekeeping professional (with an incomplete vaccination schedule), and one laboratory technician (unvaccinated) who experienced OBHs with source patients positive for hepatitis B (HBsAg positive) received immunoglobulin prophylactically, due to having anti-HBs antibody levels < 10 mIU/mL.

Regarding follow-up, according to the Ministry of Health protocol,^10^ by the end of data collection in the study period, 1.775 (72%) of HCWs were discharged, 423 (17.2%) were discharged without seroconversion among those who remained under outpatient follow-up, while 268 (10.9%) discontinued the follow-up. There were no cases of seroconversion to HBV.

## DISCUSSION

The analysis revealed that both medical and nursing staff represent the categories with the highest number of accidents, which is expected because of their direct involvement in patient care.^13-15^ The occurrence of OBHs was more prevalent among women and young adults, in line with findings from other national studies.^1,11,14,16^

The Unicamp hospital complex has two tertiary-level teaching hospitals with medical residents, and this is the professional category with the most accidents when we analyze the data separately by occupation.

This can be attributed, in part, to the nature of medical residency as a specialization process typically initiated after graduation, therefore composed of less experienced professionals. Additionally, the demanding work environment, with long working hours (60 h/week), high number of patients, and associated sleep deprivation and fatigue^17^ may further contribute to these occurrences.

In these hospitals, highly complex procedures are performed that require invasive and surgical interventions, with the use of sharp instruments, which explains the high prevalence of OBHs resulting from percutaneous exposure and with blood as the organic material involved. According to the literature, percutaneous injuries caused by contaminated sharps represent a higher risk of infection by bloodborne pathogens.^2,5,6^

Regarding the work environment, the literature highlights the following factors that increase the risks for the occurrence of OBHs: lack of training in the use of technologies; service overload; insufficient material supply; reduced health team; inadequate disposal of contaminated material; overcrowded disposal containers; haste in procedures; and limited educational programs aimed at sensitizing HCWs to adhere to standard precautions.^12,13,18^

Adherence to standard precautions is also an essential safety measure for HCWs, and their absence increases the risk of exposure to biological fluids.^2,12,13^ Although the practice of proper use of PPE is mandatory, it is not always adopted: in the present study, 11% of HCWs were not using PPE at the time of the accident.

Understanding the reasons behind HCWs’ non-adherence to PPE during care activities is important for developing prevention strategies. Among the reported factors are lack of awareness about the risks, habits, unavailability of material or inadequate sizes, work overload, lack of attention, haste, technical inability, excessive self-confidence, and stress.^12,15,19^

The low frequency of HBsAg reagent among known source cases demonstrates a lower endemicity of HBV in the region, a result similar to that found in a multicenter population-based study in which most of the Brazilian territory revealed intermediate and low endemicity for the virus infection, reflecting the high vaccination coverage resulting from the National Immunization Program.^20^

The study revealed a 98% prevalence of HCWs with complete vaccination (three doses or more) against hepatitis B. This rate was higher than the 74.9% observed by Assunção et al.^21^ in a cross-sectional study that evaluated 1,808 health workers in Belo Horizonte, state of Minas Gerais. The difference in vaccination coverage based on the level of education is noteworthy, as the percentage of unvaccinated professionals with elementary education was up to three times higher compared to that of professionals with higher education.^21^

In the present study, housekeeping personnel had lower vaccination coverage compared to other professionals and students, possibly associated with their lower schooling, as 34.4% of these HCWs had incomplete primary education.

The high coverage of complete vaccination was also higher than the rates found among health workers in the state of Bahia (59.7%)^22^ and in Florianópolis, state of Santa Catarina (64.6%).^23^

The inclusion of the anti-HBs serological status proved suitable for demonstrating the acquired immunity post-vaccination, enabling a more accurate analysis regarding the protection of HCWs against HBV.^24^ In this sense, there was a serological response with protective levels (>10 mIU/mL) in 90.9% of HCWs.

In a study conducted in India, post-vaccination immunity against hepatitis B was present in 96.5% of HCWs in a tertiary care hospital, and factors such as increasing age, time elapsed from vaccination, smoking, and obesity were associated with decreased protective immune response to HBV.^24^

Other studies have also considered it relevant to evaluate the vaccine response through anti-HBs dosage. Among them, one study conducted in Campo Grande, state of Mato Grosso do Sul,^1^ with nursing professionals, found that 63.7% were considered immunized. In the city of Botucatu, state of São Paulo,^25^ a study with housekeeping personnel reported 81.7% presenting seroconversion. Additionally, a study conducted with medical residents in a university hospital in Italy, revealed that 80% of the sample had anti-HBs titers > 10 mIU/mL.^26^

Anti-HBs antibody titers are initially higher following vaccination, and although they gradually decline over time, cellular memory ensures continued immunity.^4,27^ Due to this decline, a non-reactive anti-HBs response can be justified even among HCWs who completed the full vaccination schedule (three doses), along with the small percentage of vaccine failure. In such cases, complementary vaccination schedules are indicated to enhance the immune response, such as administering a fourth dose or using the intradermal route after two previous complete intramuscular schedules without a proper immune response.^27,28^

The question of how long protection lasts after vaccination is still being studied, as is the need for booster doses. A study conducted in Japan among medical and dental students observed an average 20% decrease in anti-HBs titer within 4 months after the third dose.^29^

In the present study, the timing of the anti-HBs serological test in relation to the vaccination dates remained unknown, as most of the notification forms did not record the vaccination administration dates.

The high adherence of HCWs to HBV vaccination and the proven immunity can be attributed to several possible reasons, including easy access to healthcare services; the vaccine’s cost-free availability; the willingness of the studied population to adopt protective measures, and the requirement for vaccination proof during enrolment or hiring by the Institution, following the Ministry of Health’s recommendations for conducting anti-HBs serological testing in HCWs.^8^ However, it is worth noting that the requirement of vaccination proof does not apply to housekeeping personnel since they are employed by third-party companies.

CECOM runs a structured vaccination program at Unicamp. For incoming students in the healthcare field, their vaccination record is analyzed, and the vaccine is provided when necessary, in addition to the anti-HBs test. The HCWs who are hired also undergo serological testing and vaccination assessment during the occupational health assessment and are referred to CECOM if vaccination is recommended.

The availability of vaccines and tests in the institution facilitates the implementation of preventive actions, thereby reducing the number of health care professionals susceptible to vaccine-preventable diseases. In China, the likelihood of HCWs completing the hepatitis B vaccination schedule was higher in workplaces where the vaccine was provided free of charge.^30^

Verification of vaccination status is essential in HCWs, especially in situations such as OBHs, because those who are not immunized and experience accidents with source cases of unknown serologies or HBsAg-positive should receive prophylactic measures such as the use of immunoglobulins.^4,10^

The limitations of the study are related to the underreporting of OBHs and the retrospective data collection, as well as the unavailability of variables that were not recorded.

## CONCLUSIONS

The results showed that vaccination remains the most important strategy for preventing HBV infection. The study revealed a high HBV vaccination coverage and proof of immunity with anti-HBs reagents among HCWs and health students who had undergone OBHs. This finding demonstrates that, despite the level of occupational exposure and accidents, most professionals were effectively protected.
